# The Problem of Confounding in Studies of the Effect of Maternal Drug Use on Pregnancy Outcome

**DOI:** 10.1155/2012/148616

**Published:** 2011-11-22

**Authors:** Bengt Källén

**Affiliations:** Tornblad Institute, Lund University, 223 62 Lund, Sweden

## Abstract

In most epidemilogical studies, the problem of confounding adds to the uncertainty in conclusions drawn. This is also true for studies on the effect of maternal drug use on birth defect risks. This paper describes various types of such confounders and discusses methods to identify and adjust for them. Such confounders can be found in maternal characteristics like age, parity, smoking, use of alcohol, and body mass index, subfertility, and previous pregnancies including previous birth of a malformed child, socioeconomy, race/ethnicity, or country of birth. Confounding by concomitant maternal drug use may occur. A geographical or seasonal confounding can exist. In rare instances, infant sex and multiple birth can appear as confounders. The most difficult problem to solve is often confounding by indication. The problem of confounding is less important for congenital malformations than for many other pregnancy outcomes.

## 1. Introduction

The golden standard in medical clinical science is the randomized double-blind study. There are, however, many situations when this method is not applicable for ethical reasons. One such situation refers to use of drugs during pregnancy and pregnancy outcome. It would be unethical to randomize sick or healthy pregnant women or women who plan pregnancy to the use of a specific drug or a placebo. Conclusions therefore have to be based on nonrandomized epidemiological studies when exposure (use of drugs) occurs spontaneously. Notably when moderate effects occur, such studies are open to criticism for many reasons, for example, bias in exposure or outcome data and difficulty in the control of confounding.

**Figure 1:**
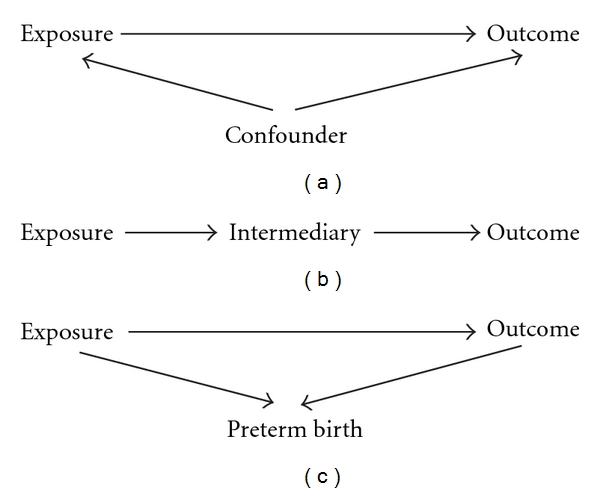
Diagrams showing relationships between exposure, outcome, and a third factor.

If one is studying the relationship between maternal use of a specific drug (e.g., an antidepressant) and the presence of, for instance, congenital malformations in the offspring an observed relationship may not be due to effects of the drug. If a factor directly affects both exposure and outcome, a confounding will exist and adjustment for it is needed. This situation is schematically shown in Figure 1(a) and can be exemplified with maternal age as a confounder in the analysis of maternal smoking and the risk for Down syndrome. In a crude analysis, maternal smoking seems to decrease the risk for Down syndrome with an odds ratio (OR) of 0.77. If, however, one adjusts for the fact that pregnant women smoke less with increasing age at delivery and that the risk for Down syndrome increases with woman's age, the OR changes to 0.94 and is far from being statistically significant. The opposite effect is obtained if the exposure is a drug, the use of which increases with maternal age, and Down syndrome is the outcome. This will result in an increased crude OR. If adjustment is made for maternal age, the effect may disappear. These two examples also show that confounding can result in a too low risk estimate or a too high estimate, depending on whether the effects of the confounder are in the opposite or the same direction on exposure and outcome.

Various methods have been used in studies on the effect of maternal drug use on pregnancy outcome. These were discussed by the author in an earlier article [1]. Many different pregnancy outcomes can be studied, for example, miscarriage, congenital malformations, preterm birth, low birth weight, intrauterine growth retardation, neonatal morbidity, and long-term morbidity including effects on neuropsychiatric development and risk of cancer. The problem of confounding will be relevant for all outcomes.

## 2. Material and Methods

Most data discussed in the paper are based on published material. Some new data are obtained by analyses of Swedish Health Registers and notably of the Swedish Medical Birth Register. Such analyses were made with Mantel-Haenszel methodology with adjustment for relevant covariables. Details of this register are available in [2].

## 3. Results and Discussion

### 3.1. Methods for the Control of Confounding

Different methods exist for the control of confounders. This can be done by matching. If the study is a case-control study, controls to cases are then selected with, for instance, the same maternal age and other characteristics one wants to adjust for. If it is a cohort study, the unexposed subjects are selected with the same characteristics as the exposed subjects. Either matching is made by selection of pairs or triplets (or more) of case and control(s) with similar characteristics, or a group of controls is chosen with a composition similar to the whole group of cases (“frequency matching”). 

More common, notably when large data sets are analyzed, is to adjust for the confounder(s) in the statistical analysis. The most common way to do this is by using a logistic regression model. This is a regression method to predict outcome (e.g., rate of congenital malformations) as influenced by one or more confounding factors. In the standard analysis, such predictions are based on linear regressions which may be inadequate, but it is obviously possible to replace them with other mathematical functions which more adequately describe the relationship between a certain variable and the outcome. (The basic formula for a logistic regression is: ln⁡(*p*/(1 − *p*)) = *α* + *β*_1_∗*X*_1_ + *β*_2_∗*X*_2_ + ⋯+*β*_*n*_∗*X*_*n*_, where ln⁡⁡(*p*/(1 − *p*)) is the logarithm for the odds of the occurrence of the outcome, *α* is a constant, and the various *β* are regression coefficients for *X*, where, for instance, *β*_1_ and *X*_1_ represent the exposure under study and *β*_2_ to *β*_*n*_ and *X*_2_ to *X*_*n*_ the various studied confounders. The regression coefficients can be transformed to odds ratios.) When the relationship is U-formed, a linear regression may not reveal the relationship properly but the fitted straight line may be nearly horizontal, indicating no relationship. There are many advantages with this method, for instance, that quantitative data do not have to be grouped and that estimates can be made by interpolations when control data are missing. It should be remembered that the effect of each one of the studied variables represents the effect after adjustment for the other included factors. If, for instance, maternal age and parity are both added in the formula, the odds ratio for maternal age will represent the effect of age adjusted for parity and *vice versa*.

Another method, especially useful when analyses are based on very large materials, is the Mantel-Haenszel technique. In this technique, cases and controls are compared within a number of strata, defined by chosen confounders, for example, maternal age group, parity group, and smoking group. Within each stratum, the occurrence of exposure among cases is compared with the occurrence of exposures among controls and a summary chi-square is calculated (based on one degree of freedom) which gives the average association between exposure and outcome after adjustment for the confounders. When this method is applied to materials of limited size, controls may be missing in some strata which are then rejected, reducing the power of the study. When large control materials are present (like in register studies) this risk is small.

The methods can of course also be used to characterize the importance of the selected putative confounders for exposure and for outcome separately and in the final analysis only true confounders (affecting both exposure and outcome) can be included. 

In most instances, the two methods give rather similar results.

Logistic regression is used in analyses aiming at risk determinations in a dichotomous situation, for instance, presence or absence of a malformation. When outcome can show a number of quantitative effects (e.g., IQ) a multiple regression model will be used instead. The adequacy of the statistical model is equally important in both methods. 

The decision on the inclusion of confounders in the final analysis can be made in different ways. It is obvious that factors which in the analysis are identified as confounders (i.e., affect both exposure and outcome) should be included. Sometimes other factors, known from previous studies or the literature as confounders, are included. It is rather common that one includes also factors which only affect exposure or outcome but not both. Such factors are thus not confounders and should not be included but if they are, it does not matter much.

### 3.2. Confounding and Stratification: The Example of Infant Sex

Confounding should be kept apart from the possibility that the effect of the exposure differs, for instance, in male and female fetuses, that infant sex modifies the effect of exposure. If this is the case, the odds ratio obtained with or without sex adjustment will be somewhere between that for male and that for female infants. This can be seen from a study of meclozine and subluxation of the hip. The odds ratio for this condition after maternal use of meclozine in male infants is 1.16 (95% CI 0.92–1.45) and in the female infant 0.83 (95% CI 0.70–0.99). These two estimates differ: *z* = 2.27, *P* = 0.03. This means that the drug (or the condition which is the indication for drug use) reduces the risk for hip subluxation in girls but not (or at least less) in boys. There exist arguments that this condition has different etiology in the two sexes. This procedure is an example of stratification in the analysis in order to see if an effect differs in subgroups of the material, in this case between boys and girls. When one makes such subgroup analyses it is important to remember that the chances for random “significances” increases with the number of subgroups studied and if differences are found one must verify that they are not due to chance (which thus seems not to be the case in example above). One way to explore such a possibility is to add, in a logistic regression analysis, an interaction term between exposure and infant sex to see if the latter factor modifies the effect of the exposure. If this interaction term is statistically significant, it means that both sex and exposure affect the outcome risk and that the effect of exposure differs according to infant sex.

An extreme situation is when outcome like hypospadias is only present in one sex. Intuitively, one would then compare exposures between infants with hypospadias and male infants without hypospadias. If exposure is independent of infant sex (which is true for most drugs), the result of only comparing with male control infants is to reduce the material which is used for estimating the background exposure rate and therefore slightly widen the confidence interval.

### 3.3. Adjusting for Intermediary Factors in the Pathway of the Effect of the Exposure on the Outcome

To adjust for a nonconfounder usually does not change the effect of the exposure—sometimes confounders are simply defined as factors which change the risk estimate with, for instance, 10% or more. There is another common situation, where adjustment may give results which can easily be misunderstood. If the exposure acts via an intermediary phenomenon which affects outcome, adjustment for the phenomenon may remove the effect more or less completely (Figure 1(b)).

We can exemplify this phenomenon from studies of neonatal conditions where the effect is more easily seen than in studies of birth defects. If maternal use of a drug increases the risk for preterm birth, any neonatal condition which occurs at an increased rate among preterm newborns will probably be increased after the exposure. If this is the only way in which the drug affects the neonatal condition, the effect will disappear after adjustment for gestational duration or a paradoxical effect can even be obtained. A classical example is that maternal smoking increases perinatal mortality but after adjustment for gestational duration or birth weight, the increased risk changes to a seemingly protective effect [3]. One will then compare infants born of smoking women (which are born preterm or with low birth weight because of the smoking but may be otherwise healthy) with infants born of nonsmoking women. Some of the latter infants are born preterm or with low birth weight because of fetal pathology and are therefore at an increased risk for perinatal death.

In such situations, the proper way of analysis is to look at the effect of exposure without considering the intermediary factor. If one wants to see, if the effect of the exposure is solely or partly due to an intermediary factor, the effect will disappear if adjustment is made for it. This may be of interest, for instance, if one wants to find out if an effect of a specific drug occurs by a direct pharmacological action on the infant or if it an effect of an intermediary, for example, an increased risk for preterm birth. 

We can exemplify this with the effect of late pregnancy use of certain CNS active drugs on neonatal morbidity (Table 1). Such neonatal morbidity is markedly more frequent in preterm than in term infants and also occurs more often in infants born by mothers using CNS-active drugs during late pregnancy than in other infants. If this had merely been an effect of preterm birth, a risk among term birth should not be seen but it is actually slightly higher than for all births.

In studies of birth defects, which originate early in the pregnancy, such intermediary effects are more difficult to detect. In this situation, another relationship may appear. Infants with some congenital malformations are born preterm more often than expected and use of a drug may increase the risk for preterm birth.  Figure 1(c) illustrates this situation. As a rule, preterm birth cannot cause the malformation, so gestational duration is no intermediary and no confounder and generally no adjustment should be made for gestational duration. The drug could, however, affect for instance the placenta, which could both increase the risk for preterm birth and for the occurrence of a congenital malformation. That such a mechanism can exist may be suggested by the observation that malformed fetuses already in mid-gestation may have an increased risk of being small for date, leading to misdating from 2nd trimester sonography [4]. The placenta effect would then be an intermediary in both causative pathways and the situation is the same as in the situation of an intermediary as discussed in the former paragraph.

### 3.4. Confounding by Maternal Characteristics

#### 3.4.1. Maternal Age

This is a classical confounder in many studies of teratogenic effects of drugs. The effect of maternal age (5-year classes) on the risk for any relatively severe congenital malformation is seen in Table 2. Note that each studied variable in this Table is adjusted for all other variables in the table in order to identify specific effects of each variable. There is a not very strong J-shaped relationship between maternal age and the risk for any relatively severe malformation. Malformations vary with respect to maternal age dependency, however. In a few, a strong increase in infants born of young women is seen. The most well-known example is gastroschisis, for which the odds ratio at age <20 in the present material is 4.51 (95% CI 2.63–7.71) and at age 20–24 it is 2.41 (95% CI 1.67–3.48), using age 25–29 as a reference. For many other malformations, the risk increases with maternal age, and for some, like trisomies, the risk increases sharply at high age. For Down syndrome, for instance, the risk increases at 30–34 years to 1.76 (95% CI 1.52–2.03) and at 40–44 years age to 6.66 (95% CI 1.94–24.4). The effect of maternal age will therefore depend on the specific malformation under study.

Often the maternal age effect on the use of drugs is strong. In Table 2, data for the use of antidepressant drugs in early pregnancy is shown as an example. The maternal age effect on drug use varies markedly with the drug type [2]. Use of antiasthmatic drugs is, for example, more prevalent among women delivering at a young age (<25 years) than among older women while antihypertensives show the opposite distribution.

If adequate adjustment for maternal age is not made, the estimated risk after maternal use of a drug may be falsely exaggerated or underestimated according to the directions of the age effect on malformation risk and on the use of the drug. For the outcome of any congenital malformation the effect is usually weak but for specific malformations it can be of greater importance.

#### 3.4.2. Maternal Parity

The definition of the parity concept differs. In this text, parity 1 means that the woman had her first child and, for instance, parity 3 that she had two previous children (which could have been twins). Sometimes parity 0 or nulliparity is used for women at their first delivery.

The risk for any relatively severe congenital malformation (Table 2) is slightly higher at parity 1 than at higher parities but there is no change of risk between parities 2 and higher parities. For most specific malformations, the parity effect is small. For esophageal and anal atresia, an increased risk at parity 1 is seen [5] but for cleft lip/palate, an increased risk at high parity seems to exist [6].

#### 3.4.3. Maternal Smoking and Use of Alcohol or Illegal Drugs in Early Pregnancy

The effect of maternal smoking on the risk for infant malformation has been much discussed. According to Table 2, there is a weak effect on any relatively severe congenital malformation with an odds ratio of less than 1.1. The effect of smoking varies according to malformation type studied [7] but is much stronger for intrauterine growth and birth weight [8]. For cleft lip/palate the increased risk after maternal smoking is rather well established while data for many other conditions are scarce. As seen in Table 2, smoking is much more prevalent among women who use antidepressants and the same is true for the use of many other CNS active drugs. The specific relationship with different drug groups can be found in [2]. One interesting such relation is with antihistamines where smoking is less prevalent than expected, notably for antihistamines used for nausea and vomiting in pregnancy (NVP). There are arguments suggesting that the relation is not explained by the fact that women with NVP stop smoking but that NVP is less prevalent among smoking women who get pregnant [9].

Adjustment for maternal smoking is thus at least sometimes needed. Such adjustment can be made using a yes/no question but a quantitative estimate is often preferable, notably when the smoking effect is strong. How detailed such a quantification should be made depends on the available possibilities. Often a division into smoking <10 cigarettes per day and 10 or more cigarettes per day is made as in Table 2. There is no convincing difference between the groups in the column for relatively severe malformations but definitely one in the column for antidepressant use.

Also the use of other nicotine preparations may occur, for example, snuffing and nicotine applications for treatment of smoking addiction. Some evidence has been presented for a teratogenic effect also at these administrations but no firm conclusions can yet be drawn and more data are needed. Hypoxia or carbon monoxide and not nicotine may cause the teratogenic action of maternal smoking [10].

In a few instances, maternal smoking appears to have a “protective” effect with a lower malformation risk among infants of smokers than of nonsmokers, for example, neural tube defects and hypospadias [7]. The mechanism behind this is unclear.

Complex addiction is common, and there is a strong association between smoking and alcohol use. The effect of the use of large amounts of alcohol on the embryo is well known and can result in a recognizable “fetal alcohol syndrome” [11] in which presence of a cardiac defect is one component. A teratogenic effect of moderate amounts of alcohol is more dubious [12]. Associations between alcohol use and some specific malformations like omphalocele and gastroschisis have been based on retrospective studies with a risk for recall bias [13]. Information on alcohol use in large numbers of individuals is difficult to get from routine questionnaires or interviews, and efforts to adjust for confounding from alcohol use are therefore often ineffective. The importance of alcohol as a confounder obviously depends on the prevalence of alcohol use and abuse among pregnant women which probably varies much between different populations. Because of a strong association between smoking and alcohol use, adjustment for smoking (which is usually easier to get reliable data on) may take care of at least part of the confounding obtained by alcohol use.

It is also difficult to get information on the use of illegal drugs, notably in populations where such use is regarded as unacceptable social behaviour. Use of many of these drugs can have important effects on pregnancy and infant morbidity but usually they are not very important confounders in studies of congenital malformations. It is true that some of these drugs have been associated with specific teratogenic effects but these studies have been based on retrospective exposure data collection with a risk for recall bias [14].

#### 3.4.4. Body Mass Index

Increasing interest is being paid to the possible impact of the ongoing obesity epidemic in many parts of the world. Many ill effects of prepregnancy obesity on pregnancy outcome has been found, including an increased risk for many (but not all) congenital malformations [15]. As is seen in Table 2, there is a clear-cut increased risk in the risk for any relatively severe malformation and obesity is also associated with a strongly increased use of antidepressant drugs. Leanness, on the other hand, in general seems not to affect malformation risk.

The mechanism behind the effect of obesity on malformation risk is unclear. A possible explanation is that obesity is associated with an increased risk for diabetes type 2 which often goes unnoticed and undiagnosed for a long time and which seems to have a teratogenic action similar to but weaker than that of diabetes type 1 [16].

Information on the two variables which define body mass index, weight and height, should refer to the time just before pregnancy or possibly to early pregnancy while weight at delivery is affected by weight changes during pregnancy which are of little interest for teratogenesis. The information can be based on anamnestic information given by the pregnant woman (with some uncertainty) or actual measurements at the first antenatal visit if this occurs early in pregnancy. As long as the information is collected before the outcome of pregnancy is known, it will be unbiased.

#### 3.4.5. Subfertility

The usual measure of subfertility is how many years the couple has tried to get a pregnancy before they succeeded. Clinically, a waiting time of less than one year is not regarded as indicating subfertility and the concept of subfertility should not be mixed with the concept “time to pregnancy,” which usually indicates the number of menstrual cycles which has passed before conception. It is known that a period of unwanted childlessness increases the risk for adverse pregnancy outcomes [17] including a moderately increased risk for infant congenital malformations (Table 2). This factor is of course especially important in studies of drugs or other treatment for infertility, including in vitro fertilization [18], but also the use of other drugs may be affected by subfertility. In Table 2 it is seen that antidepressant use is reduced at long-standing subfertility (3 years or more), a situation where various treatments including in vitro fertilization may be considered. The same phenomenon is seen for sedatives and hypnotics [2]. Under these circumstances, the women may actively try to increase the chance for conception and a healthy pregnancy by avoiding use of these drug. Use of, for instance, antihypertensives or antiasthmatics, on the other hand, is associated with an increased occurrence of subfertility [2]. This may be due to a direct effect of the underlying disease or the drug on the possibility to conceive.

#### 3.4.6. Previous Miscarriages

A previous miscarriage may increase the risk for a congenital malformation in a newborn, and this risk may increase slightly if more than two previous miscarriages have occurred (Table 2). For some conditions, the relationship can be stronger than that. This can—like threatened abortion during an ongoing pregnancy—act as an important confounder in analyses of drugs which are used to treat these conditions. Some confounding effect can also be obtained for other drugs. In Table 2, for instance, it can be seen that repeated previous miscarriages (3 or more) are associated with an increased use of antidepressants and a similar relation exists for sedatives and hypnotics [2].

#### 3.4.7. Previous Birth of a Malformed Infant

For many malformations, a genetic component is important, for example, orofacial clefts, neural tube defects, and cardiac defects. The presence of an older sibling or other close relative with such a malformation will therefore affect the risk for a malformation in a new pregnancy. This phenomenon will be a confounder in analyses of drug effects only if the birth of an infant with a malformation will affect the use of drugs in the following pregnancy. There is little information on this available. An ongoing study using the Medical Birth Register in Sweden indicates the complexity of this issue. The odds ratio for using (and reporting) a drug in early pregnancy is actually higher in women who had a malformed infant in a previous pregnancy compared to other women after adjustment for year of birth, maternal age, parity, smoking in early pregnancy, and BMI. The OR is only 1.14 (95% CI 1.08–1.21), and its size depends on the type of malformation that occurred: it is increased for neural tube defects and for cardiovascular defects but not for orofacial clefts, alimentary tract atresia, severe kidney malformations, hypospadias, or chromosome anomalies. The increased OR for neural tube defects is nearly exclusively explained by the use of folic acid. For cardiac defects, the main contribution is not only from insulin but also from psychopharmaca. 

For any relatively severe malformation, the OR varies with the drug category (Table 3). The highest OR has folic acid, insulin, psychopharmaca, thyroid drugs, and NSAID (nonsteroid anti-inflammatory drugs). Also for anticonvulsants and antihypertensives ORs are high although not statistically significant.

That folic acid is used more often in the pregnancy following a birth of a malformed infant (notably neural tube defect) is reasonably the result of a therapeutic tradition, notably the recommendation of high doses of folic acid after a pregnancy complicated with a neural tube defect. A protective effect of folic acid for cardiovascular defects has also been suggested even though evidence is less clear in that case. In the (rather few) instances when folic acid use is due to a previous malformation with a significant recurrence risk, a confounding will exist.

For some other drugs the explanation of an increased OR may be the following. If the woman has a chronic disease like diabetes type 1 which has a marked teratogenic effect, the presence of such a defect (e.g., a cardiovascular defect) in a previous pregnancy can be due to the disease. As this is a chronic condition, any following pregnancy in such women will be characterized by maternal diabetes type 1 and use of insulin. Possibly a similar relationship is seen for hypothyreosis, epilepsy, and chronic hypertonia; all these diseases and drugs used for treating them are associated with a moderately increased risk for infant cardiovascular defects. A previous child with a malformation caused by the disease will then not act as a confounder.

A third possible explanation is especially applicable to psychoactive drugs. If the burden of a handicapped or sick child increases the use of this type of drugs, women with a previous birth of a malformed infant will be more likely to use such drugs, and in this situation, the presence of a previous child with a malformation will represent a true confounder. 

There is little evidence that the birth of a previous malformed child will reduce drug use in a following pregnancy. In none of the analyses performed, an OR significantly under 1.0 was found.

A different question is if a drug effect differs between cases with and without a known genetic risk for a malformation. By identifying women who already had a pregnancy with the same malformation as in the current pregnancy product, a crude division can be made between genetic high-risk and low-risk fetuses. To explore the impact of genetics on the drug effect a stratification of the material can be made into women with and without previous pregnancies with the malformation in question [19]. Unfortunately, numbers are usually so low in the former group that the study does not become informative. One could believe that by excluding infants with a genetic load from the study, the sensitivity for the drug teratogeneic effect should increase. On the other hand, the drug may act only on a genetic background by increasing the penetrance of the genes which contribute to the origin of the malformation. 

Similarly, studies have been made where cases were divided into presence or absence of specific genes, associated with the origin of a certain malformation—again numbers have usually been so low that no firm information was obtained [20].

#### 3.4.8. Prenatal Diagnosis and Induced Abortion

Some congenital malformations can be identified by various methods applied during pregnancy, and if the malformation is regarded as serious this may result in an interruption of the pregnancy. This is a problem in data collection but could also lead to confounding. Use of a drug—or more likely the underlying disease—may affect the probability that a prenatal fetal investigation is carried out or could affect its degree of detail. This has, for instance, been suggested to occur in depressed women. This problem is most important in populations where all pregnant women do not routinely get a sonographic examination which, for instance, is the case in the Scandinavian countries.

#### 3.4.9. Socioeconomy

The importance of parental socioeconomy varies according to the pregnancy outcome investigated and the population studied. To a large extent, socioeconomic effects can be explained by lifestyle factors, for example, smoking and obesity, the effects of which can be directly controlled when such data are available. For some outcomes, some effects of socioeconomy will remain but it is doubtful to what extent this is true for birth defects. One possible pathway would be via nutrition and food quality. Such effects are probably much stronger in societies with large socioeconomic differences than in wellfare societies. This may be an explanation that socioeconomy appears to be related to neural tube defects in some countries like Great Britain [21] and USA and is hardly discernible in North European countries like Sweden.

Also the impact of socioeconomy on drug usage depends on the wellfare situation in the society. When medical care is free or associated with low costs, the impact will probably be less than in societies where the patients to a large extent have to pay for medical care and drugs. Obviously, there are also socioeconomic differences in disease rates which affect drug use.  Table 4 shows the different effects of maternal education level for some drug groups. Even though many show statistically significant deviations, the ORs are only slightly increased or decreased, but for some drug groups and notably for CNS active drugs, more marked differences are seen. To what extent these differences are due to different prevalence of underlying disease or to different drug use at similar underlying disease patterns is difficult to disentangle.

The significance of socioeconomic factors as confounders varies between populations, mainly according to the impact on malformation risk.  Table 5 indicates that, in Sweden, maternal education as a proxy for socioeconomic level has little impact on malformation rate if adjustment is made for age, parity, smoking, and BMI. For specific malformations and notably for hypospadias, an association seems to exist with a moderately increased risk at short education and a possibly lower risk at high education. This could confound an analysis, for instance, of the possible effect of hormonal treatment on hypospadias risk. For severe kidney malformations, a reduced risk is seen for infants of women with a high education. It can be noted that no effect of socioeconomic level is seen on the risk for an infant with a chromosome anomaly which indicates no difference in prenatal detection rate according to maternal education, and there is no effect on (sub)luxation of the hip, a condition sensitive to variable diagnostic and reporting completeness. The classical effect of socioeconomy on neural tube defect risk is not seen in this material.

Maternal education is one way to evaluate socioeconomic conditions. In some societies, the man's education plays a more important role than the female's for the socioeconomy of the family. Formal social group classifications exist in some countries. Family income may be a useful variable when known, but in many wellfare societies with extensive social security systems and usually also high tax rates, this measure may be less sensitive.

A further variable which can be used is if the woman is cohabiting or not with the man at the beginning of pregnancy. In Sweden only 3.3% of the women who give birth are not cohabiting in early pregnancy—this may be the result of the abortion law which permits abortions without any restrictions before week 12.  Table 5 demonstrates the weak effect of this variable on malformation risk—a “protective” effect is seen on (sub)luxation of hip which may be a result of multiple testing.

When other outcomes than congenital malformations are studied, like preterm birth and intrauterine growth retardation, socioeconomic level can have a stronger effect.

#### 3.4.10. Race/Ethnicity and Country of Birth

In many populations, race or ethnicity is an important confounder in reproduction epidemiology. Other populations are rather homogeneous from a racial point of view. In some societies information on race is not politically possible to record (e.g., in Sweden). In such areas, sometimes country of birth can give an idea of these factors but noticed effects can also be related to the status of being an immigrant. Analyses of the effect of maternal country of birth on pregnancy outcome in Sweden indicated that only few groups may deviate from Swedish-born women, among them women from Sub-Saharan Africa [22]. They will make up a rather small proportion of the studied population. 

Table 6 shows that the country of birth of the woman sometimes slightly affects the risk for an infant with a relatively severe malformation. Due to the large numbers involved, some odds ratios reach statistical significance even though the magnitude of the deviation is small. Both women born in the other Nordic countries and women born in non-Nordic countries have a slightly decreased risk for a malformation in the infant. These women have moved to Sweden for various reasons. One group (often from Asia) were adopted as children and have lived most of their life in the Swedish society and have Swedish as their native language but carry the genetic load of their country of origin. Some women were born by Swedish parents who at that time were living abroad. Other women have immigrated as refugees from catastrophe or war areas; some have immigrated for purpose of searching work (often highly educated) or because they had a relationship with a Swedish man. 

The weak tendency to a malformation risk slightly below that for Swedish-born women could perhaps be explained by a “healthy immigrant” effect, that at least some of the reasons listed above will favour women without chronic diseases.

The genetic composition will vary between geographical areas why specific conditions may be present for specific malformations. As an example different rates of neural tube defects in USA are seen when white, black, and Hispanic populations are compared [23].

The pattern of drug use during pregnancy often differs according to country of birth [2]. As a confounder, country of birth will be rather weak but when in doubt it may be wise to repeat an analysis based only on nonimmigrant women.

#### 3.4.11. Confounding by Concomitant Use of Other Drugs

Women often use combinations of drugs, and in studies of one specific drug, other drugs may act as confounders, if they are used more often together with than without the drug under study and if they in themselves increase the risk for instance of a birth defect. In Table 7 some examples are given based on maternal use of antidepressants [24]. Many drug groups are used in excess by these women. The strongest relationship is seen with oral contraceptives and psychoactive drugs: opioids, antipsychotics, and sedatives/hypnotics. Weaker but statistically significant relationships are seen with drugs for stomach ulcer and reflux, systemic corticosteroids, thyroid drugs, NSAIDs, antiasthmatic drugs, and antihistamines. Much of these associations can be explained by known comorbidity. On the other hand no associations is seen with some drug groups, and some are used less often by women taking antidepressants than by other women: multivitamins, folic acid, and minor analgesics. It can be debated if this mirror an actually lower use or is an effect of the fact that women may concentrate on reporting the use of potentially harmful drugs and neglect common and apparently harmless drugs.

Coexposure for different drug categories can have different results. In order to act as confounders, the codrug must in itself affect outcome. This may be true for some of the drugs listed but probably not for other, for example, drugs for stomach ulcer or reflux and antihistamines. The use of a nonteratogenic drug will not appear as a confounder in studies of birth defects: even if it is associated with the exposure under study as it does not associate with the outcome under study. The number of women who have been exposed to a putative teratogenic drug by coexposure is usually low, and the exclusion of such cases in the analysis may be the easiest way to deal with the problem.

There is another complication which can occur due to exposure for two drug categories. Neither of the two drug groups may have a noticeable teratogenic activity but when used together they may—they would act synergistically. This, for instance, was suggested in a study [25] indicating that neither SSRI drugs nor benzodiazepines had an observable teratogenic effect, but the combination of the two drugs had. This observation, based on rather few cases, has not yet been confirmed in an independent material.

### 3.5. Confounding by Infant Characteristics

#### 3.5.1. Infant Sex

Some malformations show a deviating sex ratio, sometimes extreme (like hypospadias which in practice only exists in males). Is there then a reason to adjust for infant sex in the analysis of drug effects?

If the use of a drug is affected by infant sex, this would lead to confounding and adjustment for sex should be made. Thus, for instance, subluxation of the hip is more common in girls than in boys (sex ratio 0.37 instead of 1.06 among all neonates) and women who carry a girl fetus are slightly more likely to experience nausea and vomiting in pregnancy (NVP) and therefore also to use drugs for that condition (for instance, antihistamines with an antiemetic effect). In a study of the use of meclozine, the infant sex ratio was 0.92 instead of 1.06 [26]. If one wants to study the possible relationship between the use of meclozine (or NVP) and occurrence of subluxation of the hip in the newborn, an adjustment for infant sex is therefore called for. Actually, this changes the odds ratio very little: from 0.95 (95% CI 0.82–1.08) to 0.91 (95% CI 0.71–1.05). 

In most situations, infant sex is unrelated to drug exposure and is therefore not confounder, even if the sex distribution among the outcome is skewed. No adjustment for infant sex is then called for. As pointed out above, infant sex may modify the effect of the drug which can be analyzed by subgroup analysis according to sex.

#### 3.5.2. Multiple Birth

The occurrence of multiple birth may be affected by drug treatments even though this situation is rare. One such example is ovulation stimulation with, for instance, clomiphene, leading to an increased rate of twin pregnancies. In most instances, drug treatment in early pregnancy does not affect the rate of multiple births. An example of a possible effect is exposure to SSRI (but not tricyclic antidepressants) when the twinning rate is significantly low [2] Some evidence exists that use of folic acid may increase the twinning rate. Most of the changes in twinning rate refer to dizygotic twins. 

The malformation rate in twin infants differs only little from that in singletons and an increased risk is mainly seen in monozygotic twins. Among dizygotic twins, some conditions associated with prematurity and perhaps with intrauterine crowding may be increased. 

In a situation when adjustment for twinning is needed, it should be remembered that the effect of drug use on twinning rate concerns dizygotic twins, and if adjustment is made for any twinning, one will underestimate the risk for malformation after the exposure because in the reference population the percentage of monozygotic pairs will be higher than in the exposed population. This was clear in studies of twins born after in vitro fertilization (IVF)—when IVF twins were compared with spontaneously conceived twins, the former appeared to show less neonatal pathology than the latter, but when comparisons were made between unlike sexed (dizygotic) pairs the IVF twins had a worse outcome than the non-IVF twins [27].

#### 3.5.3. Gestational Duration, Birth Weight, and Intrauterine Growth

Infants with birth defects are sometimes born preterm with low birth weight and signs of intrauterine growth restriction. These expressions are the result of the malformations or have a common cause with the malformations, for example, placental insufficiency. They are thus neither intermediaries, nor confounders, and there is generally no reason to adjust for them. Only when a birth defect is studied which is the result of preterm birth, gestational duration can appear as an intermediary. This could, for instance, be the case with undescended testicle and persistent ductus arteriosus, both strongly associated with preterm birth and hardly true malformations.

Birth weight strongly depends on gestational duration. If the use of a drug increases the risk of short gestational duration, it will usually also affect birth weight as an intermediary between exposure and outcome and gestational duration should not be adjusted for when one is interested in the effect on birth weight. If one makes such adjustments, a remaining effect on birth weight will indicate that the weight of the infant at each gestational week deviates from the expected weight, which is reasonably an effect of a disturbed intrauterine growth, resulting in small-for-gestational age (SGA) or large-for-gestational age (LGA) infants. These are often interesting outcomes but they can be studied more directly.

### 3.6. Geographical and Seasonal Confounding

**Figure 2:**
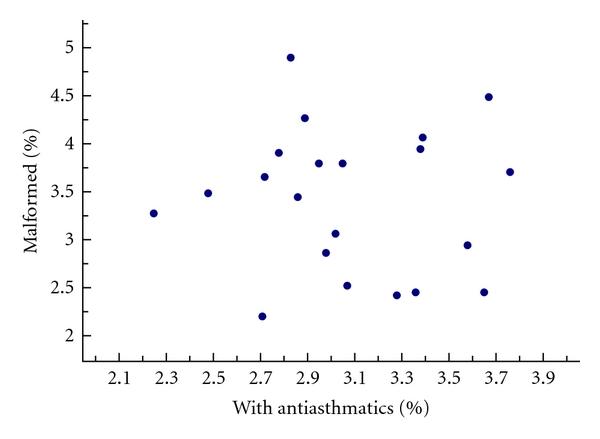
Percentage of infants with relatively severe malformations and percentage of women who used antiasthmatics in early pregnancy in 21 counties in Sweden (2000–2008).

A confounding situation can occur due to an uneven distribution of both drug use and the occurrence or completeness of registration of birth defects. Geographical confounding will be most pronounced when the studied population is distributed over a large area where variations in disease rates and/or in therapeutic traditions may occur and where also occurrence or registration of birth defects may vary. These two sources of variation are usually independent, but if they covary, a confounding can arise. Such an analysis is shown in Figure 2 which compares rates of infants with relatively severe malformations (varying between 2.25 and 3.76%) and rates of women who reported the use of antiasthmatic drugs in early pregnancy (varying between 2.20 and 4.89%) in 21 Swedish counties. A correlation analysis gives *r* = 0.13, *P* = 0.57, which suggests that the two variables do not covary significantly. 

**Figure 3:**
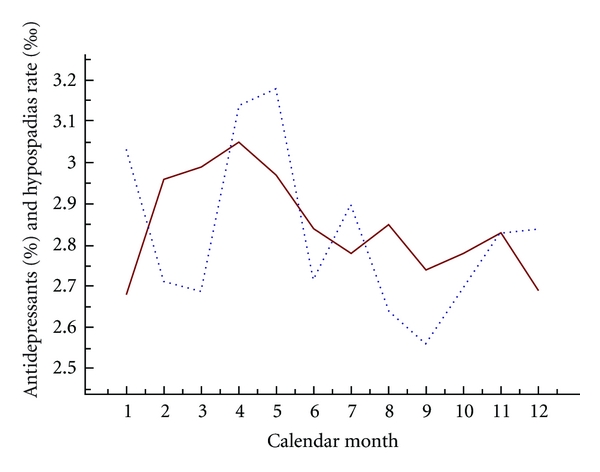
Rates of antidepressant use (percentage, unbroken line) and of hypospadias (per thousand, broken line) for the different calendar months.

Sometimes there is a clear seasonality in drug use. A typical example is drugs for allergy which at least in Northern Europe shows a peak of use during spring. A similar peak is also seen in the use of antidepressants (Figure 3). If also the malformation studied shows a seasonality, a confounding may arise if the effect of such drugs is studied. Obviously, it is not the seasonality at birth which is of interest but the seasonality at the formation of the malformation. An example is a possible association between use of SSRI and hypospadias [24] which could tentatively be due to the fact that use of antidepressants in early pregnancy may coincide with a peak of hypospadias in infants conceived during spring. The seasonality of hypospadias is rather weak but there is a peak among infants conceived during April and May which corresponds to the peak in antidepressant use (Figure 3). The correlation between the monthly rate of antidepressant use and the monthly rate of conceptions leading to hypospadias is, however, rather weak and not statistically significant (*r* = 0.26, *P* = 0.20), and this correlation is still weaker if the formative period of hypospadias (8 weeks or more after LMP) is considered instead.

### 3.7. Confounding by Indication

The strongest and most difficult to control confounding is confounding by indication: that the disease or complaint which is the reason for drug use in itself affects pregnancy outcome. 

The use of insulin during early pregnancy is associated with an increased risk for many types of congenital malformations. As insulin is practically only used at diabetes and a pregnant woman with diabetes type 1 always gets insulin, it is theoretically impossible to separate the effect of disease and treatment. As clinical experience indicates that strict blood suger control in pregnant diabetic women is important for the success of the pregnancy and also probably reduces the birth defect risk, it is generally accepted that the increased malformation risk is due to diabetes and not to insulin even if it can be debated if strict scientific evidence for this exists.

In most instances, the degree of overlap between disease and drug use is not so strong but to disentangle the contribution of the drug and the underlying condition is often difficult. We will take a number of examples to illustrate the dilemma.

#### 3.7.1. Epilepsy and Anticonvulsants

The first study that linked epilepsy with an increased risk for a birth defect concerned orofacial clefts [28] and could not distinguish between disease and treatment. Numerous studies have verified that the relationship between epilepsy and birth defects includes many different malformations, for example, spina bifida, cardiovascular defects, orofacial clefts, hypospadias, that the effects of different anticonvulsants vary, and that untreated epilepsy seems not to be associated with an increased birth defect risk.

Anticonvulsants are also used for other medical conditions than epilepsy, for example, as mode stabilizers at bipolar disease and sometimes for neuropathic pain (e.g., gabapentin). No large enough studies have been published to evaluate if such use carries a similar risk as when the drugs are used at epilepsy.

The general opinion is that anticonvulsant drugs *per se* represent a teratogenic risk, and notably for valproic acid this risk can be large why such use should be avoided during pregnancy.

#### 3.7.2. Depression and Antidepressants

Antidepressants have in general a low teratogenic potential but some data indicate that tricyclic antidepressants (TCAs) carry a higher birth defect risk than selective serotonin reuptake inhibitors (SSRIs) do, while data for serotonin/noradrenalin inhibitors (SNRIs) are still incomplete. The teratogenicity of TCA is notably evident for cardiovascular defects—a similar effect is indicated in some studies for paroxetine but may be absent for other SSRIs [29] while other studies have found also an effect of fluoxetine [30].

More common effects are seen after antidepressant use in late pregnancy, resulting in preterm birth and increased risks for various forms of neonatal morbidity. In this situation, the importance of the effect of the underlying disease, usually depression, has been much discussed. Some studies describe such effects associated with maternal depression but it is not always clear if consideration has been taken to drug treatments.

#### 3.7.3. NVP and Antihistamines

A classical example of confounding by indication is the use of certain antihistamines for the treatment of nausea and vomiting in pregnancy (NVP). Studies of such antihistamines have shown a lower than expected rate of birth defects in the offspring and also other signs of a better than expected pregnancy outcome [26]. It does not seem likely that the drugs actually prevent the occurrence of birth defects, and there is some evidence that the strength of NVP is a factor which positively correlates with pregnancy outcome. The probable mechanism is that among the factors causing NVP are hormones produced from the placenta, so a strong NVP (perhaps needing drug therapy) may indicate a well functioning placenta and thereby a decreased risk for a congenital malformation or other pregnancy complications.

#### 3.7.4. Hypertension and Antihypertensives

Some studies have associated the use of antihypertensive drugs in early pregnancy with an increased risk of birth defects and notably cardiovascular defects. The first major study found such a link for ACE-inhibiting drugs [31] but a later study [32] found no difference between such drugs and other antihypertensives in the risk for birth defects. Little is known about the effect of untreated essential hypertonia on birth defect risk but the possibility that the drug effect is due to confounding by indication has been raised.

#### 3.7.5. Infections and Antibiotics

Most antibiotics have no apparent teratogenic effects in man but in a few such associations have been suggested, either from epidemiological studies (e.g., erythromycin [33]) or from pharmacological considerations (e.g., trimethoprim which has a folic-acid antagonistic effect [34]). Among infections, most interest has been shown in viral infections because of the well-known teratogenic effect of rubella and some other viral infections. Viral infections are no reason for antibiotic use but undoubtedly such treatment is often given anyway. It is also possible that adequate antibiotic treatment is given for a secondary infection following a virus infection which could have increased the birth defect risk. Secondary effects of the infection can also be considered, for example, the possible harmful effects of high fever [35].

#### 3.7.6. How to Deal with the Problem of Confounding by Indication?

One straight forward way is to compare women treated with drugs with women not treated with drugs but with the same disease. A classical example is anticonvulsants and epilepsy where untreated epilepsy repeatedly has been shown to have no detrimental effect on the embryo [36]. Epilepsy is a heterogeneous disease with very variable severity and it is therefore likely that the disease panorama is different among untreated and treated women. Certain other diseases are so severe that treatment is always needed, for example, diabetes type 1, and untreated patients are nearly impossible to find.

A second possibility exists when various drugs can be used at the same underlying disease. An example is the use of SSRI drugs at maternal depression. Among the four main SSRI drugs used in Sweden, a significant difference was seen between the effect of paroxetine and the other SSRI drugs on cardiovascular defects (Table 8) [24]. This approach, however, is complicated by the fact that SSRI drugs are used at many different conditions other than depression. One such indication is anxiety and panic disorders where a special drug, for example, paroxetine, may be favoured. So there may still remain a confounding by indication. Detailed information on the indication for use may be difficult to get in a study large enough for the detection of teratogenic effects on specific malformations. Also given the same underlying disease, differences in severity could be related to drug selection. This, for instance, could explain the difference in teratogenic effects of tricyclic antidepressants and SSRI drugs [24].

Another example of the use of two alternative drugs for similar (although not quite identical) reasons is erythromycin and phenoxymethyl penicillin. The former drug but not the latter was associated with an increased risk for a cardiovascular defect [33].

In order to characterize disease status and severity, various clinical measurements can be used. In small studies this can be based on questionnaire or interview information aimed at such a characterization, ideally performed prospectively before the outcome of the pregnancy is known. Examples are studies based on information from teratology information services where, at the contact with the woman who seeks advice, specific questions on the reason for the drug use can be made and also some standardized quantification of disease severity. Unfortunately such projects usually result in rather small numbers of exposed infants, and for studies of birth defect risks, they would most likely be strongly underpowered. The same is true for specific research projects based on data from one or a few clinics dealing with a specific group of diseases, for example, centres for the treatment of epilepsy. In retrospective case-control studies, a memory bias can be obtained not only on drug use, but also on disease characterization.

Most studies on birth defect risks which are powered to detect moderate risk increases must utilize register information where a recording of detailed disease histories is difficult. One way to solve the problem is to use a case-control design and base disease evaluations on medical records, prepared before the pregnancy outcome was known, if such records can be retrieved and are reasonably well standardized. 

Another possibility which has been used for instance in studies by SSRI drugs [37] is to characterize disease and disease severity by available health data, for example, number of visits to doctors for specific reasons. Based on such information a propensity score can be built for each patient which makes it possible to match or adjust for disease severity. The efficiency of this method depends on the selection of variables used for the propensity scoring. Often information from the time before the pregnancy is used which may not be a valid scoring for the patient in the beginning of the pregnancy when birth defects are formed.

### 3.8. How Effective Is an Adjustment for Confounding?

 Whatever the technique of identification and adjustment for confounding which has been used, the question of its effectiveness remains. The situation is rather simple if an observed effect disappears when a true confounder has been taken into consideration, like in the examples of smoking or drug use and the birth of an infant with Down syndrome (see the previous). It is then reasonable to conclude that no direct effect of the exposure has been demonstrated. If a residual effect of the exposure remains, however, this can be due to incomplete identification or adjustment for the confounder(s). Some examples will be discussed.

In most instances, the confounding effect of maternal age is moderate and adjustments based on 5-year maternal intervals will be adequate. When very strong effects are seen of maternal age, notably in the lower or upper end of the age range, more exact maternal age adjustments, for example, based on one-year intervals, may be needed. Examples are the steeply increasing risk for a Down infant birth with high maternal age and a steeply increasing risk for an infant with gastroschisis with low maternal age. In both these examples, the regression between age and outcome is nonlinear, that is why a linear regression analysis may underestimate maternal age as a risk factor.

If maternal smoking appears as a risk factor, adjustment for any smoking may be insufficient and so may a crude division into <10 and ≥10 cigarettes per day. In the latter group will be included both women who smoke 10 cigarettes per day and women who smoke 20 or more cigarettes per day, and the proportion of these groups may well vary between women who have used for instance psychoactive drugs and women who have not used such drugs. If the adjustment for smoking results in a reduction of the risk estimate, one should consider the possibility that the remaining estimate may be too high due to crude information on smoking. A similar effect can occur at adjustment for BMI, if only crude groups are used.

An adjustment for the use of any other drug than the drug under study may in a similar way be insufficient if the patterns of drug use differ. A substantial percentage of women using antidepressants also use sedatives while adjustment for any other drug use will ineffectively adjust for the possible effect of the sedatives.

### 3.9. The Effect of Adjusting for Confounding on Risk Estimates

The effects of confounders on outcome vary according to the nature of exposure and outcome. The most important effects should be expected in analyses of drugs which are used at different rates in different groups of women, for instance, different age groups. The confounding variables must, however, also affect outcome in order to be relevant. Most variables studied in Table 2 had only moderate effect on the risk for any relatively severe malformation, that is why the effect of the clear differences in the use of antidepressants in early pregnancy will be weak. In Table 9 it is also seen that the stepwise adjustment for some of these variables does not markedly change the risk estimate for a cardiac malformation after maternal use of a tricyclic antidepressant in early pregnancy: the estimates vary around 1.6 and 1.7, and the crude estimate is close to the estimate after adjustment for year of delivery, maternal age, parity, smoking in early pregnancy, and prepregnancy BMI.

The table also shows similar stepwise adjustments for risk estimates of preterm birth after maternal use of antidepressants, and here the adjustment reduces the excess risk with nearly one-third, from 60% increased risk to 42% increased risk, and the strongest effects are seen from maternal smoking and obesity.

In special circumtances, the significance of confounding becomes very important. One example is a recent study on the risk for infants conceived after IVF to develop drug-treated ADHD [38]. A summary is given in Table 10. The crude OR indicates a statistically significant protective effect, that IVF children have a lower risk for this condition than other children. As in all studies of long-term effect, year of delivery can be an important confounder if exposure rate (in this case use of IVF) increases during the observation period while the follow-up time of the children decreases. Adjustment for year of delivery increases the OR but it is still significantly below 1.0. Adjustment for some maternal characteristics including age, parity, smoking, country of birth, and (in the next step) BMI removes the apparent protective effect and leaves an OR close to 1.0. As low maternal education is associated with an increased risk for infant ADHD, and high education with a decreased risk, adjustment for maternal education increases the risk estimates but it does not quite reach statistical significance (lower CI is 0.99). Also non-cohabitation in early pregnancy is associated with an increased risk for ADHD and when these (relatively few) women are removed from the analysis, the OR increases further and becomes statistically significant. What we see here is the opposite effects of some variables on exposure (IVF) and outcome (drug-treated ADHD) which initially results in an apparent protective effect which gradually disappears when such factors are added in the analysis and finally results in an apparent over-risk. It should be stressed that some of these factors (e.g., low maternal education and not cohabiting) reasonably have no direct effects but may be proxies for two things: socioeconomic level and possibly parental signs of ADHD with a genetic background. So, for instance, some studies indicate that the increased risk for ADHD if the mother smoked is due to the fact that women with genes for ADHD and perhaps have signs of ADHD smoke more than other women [39, 40]. The basic question if infants conceived by IVF do have an increased risk for ADHD is not definitely solved. This may be a good example of how complex and difficult to handle confounding can be under certain circumstances.

## 4. Concluding Remarks

The problem with confounding is not solved by uncritically adding a number of variables to a logistic regression model. Variables used for adjustment should be carefully selected according to their properties to affect both exposure rate and outcome rate. It should be realized that efforts to adjust for them may be ineffective because the available information on the confounders may be too crude or the statistical models used may be inadequate. It should be realized that the major problems in epidemiological studies of the effects of maternal drug use on birth defects are to be found in biased data and low statistical power, not so much in confounding. For studies of other pregnancy outcomes like preterm birth, low birth weight, neonatal and long-term morbidity, confounding plays a more important role. The most difficult confounding refers to confounding by indication, often stated as important but usually without any offer of a good solution for its elimination.

##  Conflicts of Interests

The author declares no conflicts of interest.

##  Ethical Considerations

The analyses were performed within the responsibilities of the National Board of Health and Welfare and therefore no ethical approval from outside ethical committees was needed.

## Figures and Tables

**Table 1:** Risk of neonatal morbidity^a^ according to preterm birth and maternal use of CNS-active drugs^b^ after the first trimester (both 2nd and 3rd trimester). Among all infants, 6.0% were born preterm, and among infants of women using CNS-active drugs, 8.2% were born preterm.

Infant group	With neonatal pathology	Total number	%	OR	95% CI
All infants	22015	315975	7.0	1.00	Reference
Preterm births	7465	18836	39.6	12.3	12.0–12.7
CNS-active drugs, all infants	541	4425	12.2	1.83	1.67–2.00
CNS-active drugs, term infants	380	4009	9.5	2.05	1.84–2.28

^a^ Infant morbidity consists of one or more of the following conditions: respiratory disorders (ICD-10 codes P22–P28), hypoglycaemia (P70.4–P70.9), neonatal convulsions (P90), other disturbances of cerebral status (P91), low Apgar score (Apgar 5 minutes <7).

^b^ The drugs studies include opioids, anticonvulsants, antipsychotics, sedative/hypnotics, and antidepressants.

**Table 2:** Impact of various maternal variables on the risk for any relatively severe congenital malformation in the infants and on the use of antidepressant drugs.

	Relatively severe malformation^a^		Maternal use of antidepressants	
Variable	OR	95% CI	OR	95% CI
*Maternal age*				
<20	1.07	0.99–1.15	0.69	0.60–0.79
20–24	1.04	1.00–1.07	0.91	0.86–0.97
25–29	1.00	Reference	1.00	Reference
30–34	1.00	0.97–1.02	1.19	1.14–1.24
35–39	1.02	0.99–1.06	1.52	1.99–1.60
40–44	1.15	1.08–1.23	1.78	1.62–1.96
≥45	1.02	0.74–1.41	2.36	1.60–3.49

*Parity*				
1	1.00	Reference	1.00	Reference
2	0.89	0.87–0.91	0.67	0.64–0.70
3	0.90	0.87–0.93	0.85	0.80–0.89
≥4	0.89	0.85–0.93	0.84	0.78–0.90

*Smoking*				
None	1.00	Reference	1.00	Reference
<10 cigs/day	1.06	1.03–1.11	2.39	2.27–2.51
≥10 cigs/day	1.09	1.03–1.15	3.84	3.63–4.07

*Body mass index*				
<19.8	1.00	0.96–1.04	1.03	0.96–1.20
19.8–25.9	1.00	Reference	1.00	Reference
26–29.9	1.09	1.06–1.11	1.22	1.17–1.27
30–39.9	1.15	1.11–1.20	1.49	1.41–1.57
≥40	1.39	1.23–1.56	2.19	1.92–2.50

Number of years of unwanted childlessness				
0	1.00	Reference	1.00	Reference
1	1.02	0.95–1.09	0.89	0.88–0.99
2	1.08	1.01–1.15	0.90	0.81–1.01
3	1.12	1.02–1.29	0.66	0.56–0.78
4	1.30	1.17–1.45	0.80	0.65–0.98
≥5	1.30	1.25–1.41	0.82	0.71–0.95

Number of previous miscarriages				
0	1.00	Reference	1.00	Reference
1	1.05	1.02–1.05	0.99	0.95–1.04
2	1.05	0.98–1.11	1.08	0.99–1.17
≥3	1.12	1.02–1.32	1.15	1.01–1.30

^a^ Any congenital malformation with the exception of the following conditions which are common and variably registered: preauricular tag, patent ductus at preterm birth, undescended testicle, hip (sub)luxation, tongue tie, single umbilical artery, nevus.

**Table 3:** Use of drugs in early pregnancy among women with a previous relatively severely malformed infant compared with women who had no known previous such malformed infant. Odds ratio (OR) with 95% confidence interval (95% CI) adjusted for year of delivery, maternal age, parity, smoking in early pregnancy, and BMI.

Drug group	Among women with previous malformed infant	Among all women	OR	95% CI
Any drug	2078	290480	1.14	1.08–1.21
Drugs for stomach ulcer and reflux	53	6062	1.17	0.89–1.55
Insulin	43	2614	2.46	2.81–3.32
Multivitamins	218	56200	0.99	0.86–1.15
Folic acid	276	43780	1.26	1.12–1.43
Antihypertensives	35	3147	1.39	0.99–1.94
Thyroid drugs	104	10728	1.31	1.07–1.59
Antibiotics	186	19036	1.04	0.89–1.20
NSAID	127	13047	1.27	1.06–1.52
Opioids	38	3974	1.18	0.85–1.63
Minor analgesics	449	51043	0.96	0.87–1.06
Anticonvulsants	20	2037	1.44	0.92–2.26
Antipsychotics	26	1992	1.53	1.03–2.39
Sedatives/hypnotics	32	3301	1.21	0.91–1.83
Antidepressants	95	11714	1.26	1.02–1.55
Any psychopharmacon	137	15558	1.29	1.08–1.53
Drugs for rhinitis	67	9944	0.86	0.68–1.10
Antiasthmatic drugs	180	22702	1.07	0.92–1.25
Antihistamines	300	38689	0.98	0.87–1.10

**Table 4:** Importance of maternal education level in Sweden for the use and/or reporting of various categories of drugs in early pregnancy [2]. Nine years of education is compulsory. Most women have 12 years of education which is used as the reference group. Adjusted for year of delivery, maternal age, parity, smoking, and BMI.

	Low education, <12 years of education		High education, ≥14 years of education	
Drug group	OR	95% CI	OR	95% CI
Drugs for ulcer and gastrointestinal reflux	1.20	1.08–1.53	0.81	0.75–0.88
Multivitamins and minerals	0.92	0.66–1.29	1.11	0.89–1.38
Anticoagulants	1.04	0.81–1.33	0.96	0.81–1.14
Haemostatics	1.42	1.01–2.00	1.08	0.85–1.38
Antihypertensives	0.85	0.71–1.00	0.82	0.73–0.92
Oral contraceptives in early pregnancy	0.88	0.87–1.02	0.72	0.62–0.83
Systemic corticosteroids	0.86	0.72–1.01	1.03	0.92–1.15
Thyroxine substitution	0.91	0.82–1.01	0.91	0.84–0.97
Antibiotics	0.90	0.85–0.95	1.07	1.03–1.11
Antivirus drugs	0.84	0.85–1.37	1.35	1.08–1.69
Vaccines	0.92	0.62–1.35	1.53	1.26–1.86
NSAID	0.90	0.83–0.97	0.87	0.82–0.92
Analgesics	1.04	1.01–1.08	0.93	0.91–0.96
Drugs for migraine	0.92	0.76–1.11	0.85	0.75–0.97
Anticonvulsants	1.49	1.26–1.76	0.71	0.61–0.82
Antipsychotics	1.52	1.17–1.99	0.97	0.93–1.28
Sedatives/hypnotics	1.75	1.56–1.96	0.86	0.76–0.98
Antidepressants	1.48	1.35–1.61	0.78	0.72–0.85
Drugs used for malaria prophylaxis	0.87	0.55–1.37	2.08	1.08–2.57
Antiasthmatics	0.96	0.91–1.02	0.90	0.97–0.99
Antihistamines used for allergy	0.77	0.70–0.82	1.11	1.05–1.17
Ophthalmics	0.63	0.47–0.86	1.35	1.17–1.56

**Table 5:** Importance of maternal education or non-cohabitation for occurrence and diagnosis of congenital malformations in Sweden. Odds ratios (ORs) with 95% confidence intervals (95% CI) for various malformation groups at different maternal education levels. For educational level, the reference is 12 years of education, for non-cohabitation, the reference is cohabitation. Adjustment for year of birth, maternal age, parity, smoking, and BMI.

	<12 years of education		≥14 years of education		Non-cohabiting in early pregnancy	
Malformation group	OR	95% CI	OR	95% CI	OR	95% CI
Relatively severe malformations	1.05	1.00–1.10	0.97	0.93–1.00	0.99	0.95–1.06
Chromosome anomalies	1.04	0.86–1.26	0.96	0.84–1.10	0.99	0.75–1.29
Neural tube defects	0.75	0.40–1.16	0.97	0.73–1.30	1.49	0.89–2.48
Orofacial clefts	0.99	0.81–1.21	0.89	0.77–1.03	0.95	0.71–1.26
Cardiovascular defects	1.02	0.94–1.61	0.97	0.87–1.08	1.08	0.97–1.22
Severe kidney malformation	1.07	0.78–1.46	0.98	0.86–1.13	0.99	0.62–1.57
Hypospadias	1.24	1.06–1.45	0.78	0.62–0.98	0.85	0.66–1.10
Pes equinovarus	0.86	0.69–1.07	0.94	0.69–1.26	1.00	0.75–1.33
(Sub)luxation of hip	0.90	0.80–1.01	0.98	0.64–1.49	0.76	0.63–0.91
Craniostenosis	0.75	0.53–1.07	1.01	0.68–1.51	0.94	0.58–1.54

**Table 6:** Importance of the mother's country of birth on the occurrence of relatively severe malformations in the infant. Odds ratios adjusted for year of birth, maternal age, parity, smoking, and BMI. Reference is infants born by mothers born in Sweden. Data for years 2000–2008.

Geographic area	Number of infants	% of all infants	% malformed infants	OR	95% CI
Sweden	727166	81.9	2.7	1.00	Reference

Other Nordic countries	16612	1.9	2.5	0.87	0.79–0.96

Western Europe, Northern America, Australia, New Zealand	10504	1.2	2.6	0.96	0.85–1.08

Eastern Europe and former Soviet Union	25855	2.9	2.5	0.89	0.83–0.97

Sub-Saharan Africa	15488	1.7	2.7	0.97	0.88–1.08

North Africa and Middle East	54186	6.1	2.9	1.04	0.98–1.09

Asia	27405	3.1	2.2	0.82	0.75–0.89

South and Middle America	10373	1.2	2.4	0.84	0.74–0.96

All non-Nordic countries	143811	16.2	2.6	0.94	0.91–0.98

**Table 7:** Concomitant drug use in early pregnancy among 11,181 women who used antidepressants [2]. Odds ratios (ORs) with 95% confidence intervals (95% CI) for use of specific group categories in women using antidepressants compared with women who did not. Adjustment for year of birth, maternal age, parity, smoking in early pregnancy, and BMI.

Drug group	Number of users	OR	95% CI
Drugs for stomach ulcer and reflux	316	2.92	2.61–3.26
Drugs for inflammatory bowel disease	41	1.27	0.93–1.73
Insulin	49	1.13	0.85–1.50
Multivitamins	559	0.78	0.72–0.85
Folic acid	519	0.82	0.75–0.90
Oral contraceptives during pregnancy	160	3.52	3.02–4.11
Gonadotropins	22	0.85	0.56–1.28
Systemic corticosteroids	65	1.55	1.21–1.98
Thyroid drugs	317	1.89	1.69–2.11
Antibiotics	321	0.92	0.82–1.03
NSAIDs	300	1.21	1.08–1.36
Opioids	261	3.48	3.09–3.93
Minor analgesics	835	0.83	0.77–0.89
Anticonvulsants	110	3.17	2.63–3.82
Antipsychotics	283	7.13	6.39–7.97
Sedatives, hypnotics	1202	25.9	24.6–27.3
Drugs for rhinitis	146	0.92	0.78–1.09
Antiasthmatics	533	1.40	1.28–1.53
Antihistamines	1097	1.79	1.68–1.90

**Table 8:** Association between maternal use of SSRI drug in early pregnancy and occurrence of cardiovascular defects in the infant [21]. Odds ratio (OR) with 95% confidence intervals (95% CI) adjusted for year of birth, maternal age, parity, smoking, and BMI. The cardiovascular defect rates in the four SSRI groups differ significantly: *χ*_(3  *d*.*f*)_^2^ = 12.5, *P* < 0.01.

SSI drug	Number of cardiovascular defects	OR	95% CI
Fluoxetine	21	1.31	0.85–1.02
Citalopram	37	0.86	0.62–1.20
Paroxetine	24	1.66	1.09–2.53
Sertraline	26	0.74	0.50–1.09

**Table 9:** The effect of adjustment for some maternal characteristics on the effect of tricyclic antidepressants (TCAs) on the occurrence of infant cardiac defect and on the effect of antidepressants on preterm birth.

	Cardiac defects after TCA		<37 weeks after antidepressants	
Variables adjusted for	OR	95% CI	OR	95% CI
None (=crude)	1.64	1.14–2.36	1.60	1.50–1.72
Year of delivery	1.75	1.22–2.52	1.60	1.44–1.77
Year of delivery and maternal age	1.74	1.21–2.50	1.58	1.40–1.79
Year of delivery and maternal age and parity	1.74	1.21–2.50	1.54	1.37–1.77
Year of delivery and maternal age and smoking	1.72	1.19–2.49	1.44	1.23–1.69
Year of delivery and maternal age and smoking and BMI	1.68	1.15–2.45	1.42	1.32–1.52

**Table 10:** Effect of stepwise adjustment for maternal characteristics in the analysis of the risk for drug-treated ADHD in infants conceived by IVF [34].

Variables adjusted for	OR	95% CI
None (=crude)	0.71	0.62–0.81
Year of delivery	0.77	0.68–0.87
Year of delivery, maternal age, parity, smoking and country of birth	0.91	0.81–1.05
Year of delivery, maternal age, parity, smoking, country of birth, and BMI	0.95	0.83–1.08
Year of delivery, maternal age, parity, smoking, country of birth, and education	1.14	0.99–1.31
Year of delivery, maternal age, parity, smoking, country of birth, and education, with exclusion of non-cohabiting women	1.18	1.05–1.36
